# Supplementary material for: Trajectories of inflammatory biomarkers over the eighth decade and their associations with immune cell profiles and epigenetic ageing
Source: Clin Epigenetics. 2018 Dec 20;10:159. doi: 10.1186/s13148-018-0585-x (PMC6302523; doi:10.1186/s13148-018-0585-x)
Supplement: Supplementary file 1 — Table S1. Baseline associations between (low-sensitivity) C-reactive protein ≤10 mg/L and imputed immune cell profiles. (DOCX 14 kb) [file 13148_2018_585_MOESM1_ESM.docx]

**Additional file 1.**

**Table S1.** *Baseline associations between (low-sensitivity) C-reactive protein ≤ 10mg/L and imputed immune cell profiles*.

|  | *Standardised beta* | *SE* | *P* |
| --- | --- | --- | --- |
| *Total CD8+T* | -0.052 | 0.035 | 0.139 |
| *Total CD4+T* | -0.044 | 0.036 | 0.209 |
| *Naïve CD8+T* | -0.118 | 0.035 | **0.0009** |
| *Naïve CD4+T* | -0.027 | 0.035 | 0.445 |
| *B cells* | 0.007 | 0.035 | 0.831 |
| *Natural Killer* | -0.015 | 0.035 | 0.660 |
| *Senescent CD8+T* | 0.102 | 0.035 | **0.003** |
| *Monocytes* | -0.027 | 0.036 | 0.445 |
| *Plasmablasts* | 0.076 | 0.035 | **0.036** |
| *Granulocytes* | 0.038 | 0.035 | 0.261 |

Significant associations are highlighted in bold.
