# Supplementary material for: Trajectories of inflammatory biomarkers over the eighth decade and their associations with immune cell profiles and epigenetic ageing
Source: Clin Epigenetics. 2018 Dec 20;10:159. doi: 10.1186/s13148-018-0585-x (PMC6302523; doi:10.1186/s13148-018-0585-x)
Supplement: Supplementary file 2 — LBC1936 DNA Methylation. DNA methylation profiling methods of LBC1936. (DOCX 17.9 kb) [file 13148_2018_585_MOESM2_ESM.docx]

**Additional file 2.**

**LBC1936 DNA Methylation**

Detailed information on the DNA methylation profiling of LBC1936 has been reported previously [1, 2]. Briefly, DNA methylation was measured at 485,512 CpG sites from whole blood samples using the Illumina HumanMethylation450 BeadChips. Quality control analysis was performed to remove probes with a low detection rate (<95% at p<0.01), low-quality (inadequate hybridisation, bisulfite conversion, nucleotide extension and staining signal) and low call rate (<450,000 probes detected at p<0.01). Those samples where predicted, and reported, sex did not match, and probes on the X and Y chromosomes were additionally removed leaving a total of 450,276 autosomal probes. DNA methylation samples were processed at three separate time points (Table 1). This time point variable was denoted as 'set' and was included as a random effect in the main analyses.

|  | *Set 1* | *Set 2* | *Set 3* | *Total* |
| --- | --- | --- | --- | --- |
| *Wave 1* | 872 | 34 | x | 906 |
| *Wave 2* | 261 | 535 | 5 | 801 |
| *Wave 3* | 237 | 382 | x | 619 |
| *Wave 4* | *x* | x | 507 | 507 |

**Table 1.** *Number of methylation samples processed at each time point (set).*

**References**

1. Shah, S., et al., *Genetic and environmental exposures constrain epigenetic drift over the human life course.* Genome Res, 2014. **24**(11): p. 1725-33.

2. Marioni, R.E., et al., *DNA methylation age of blood predicts all-cause mortality in later life.* Genome Biology, 2015. **16**(1): p. 25.
